# Real world data with concurrent retinoic acid and arsenic trioxide for the treatment of acute promyelocytic leukemia

**DOI:** 10.1038/s41408-022-00619-3

**Published:** 2022-01-31

**Authors:** Uday P. Kulkarni, Sushil Selvarajan, Sharon Lionel, Mithun A. Prakash, Hamenth Kumar Palani, Nithya Balasundaram, Arvind Venkataraman, Anu Korula, Anup J. Devasia, N. A. Fouzia, Nancy Beryl Janet, Sukesh Chandran Nair, Aby Abraham, Thenmozhi Mani, Jeyaseelan Lakshmanan, Arun Kumar Arunachalam, Poonkuzhali Balasubramanian, Biju George, Vikram Mathews

**Affiliations:** 1 Department of Haematology, Christian Medical College, Vellore, India; 2 Department of Immunohaematology and Transfusion Medicine, Christian Medical College, Vellore, India; 3 Department of Biostatistics, Christian Medical College, Vellore, India

**Keywords:** Chemotherapy, Acute myeloid leukaemia

**Dear Editor**,

Multiple studies have established the synergy between ATO and ATRA in APL, and a combination of these drugs without chemotherapy is the new standard of care in low and intermediate-risk patients [1–5]. Post induction, ATRA was administered in two weeks on two weeks off schedule in these studies while ATO was administered continuously for 4 weeks on and off. The rationale for the short pulses of ATRA, post-induction, are based on the concerns of induction by ATRA of cellular retinoic acid-binding proteins I and II (CRABPI/II), acceleration of its own catabolism by induction of cytochrome P450 enzymes. However, it must be noted that while prolonged administration reduces peak and mean plasma ATRA [6], these trough are still reported to be above the critical level of 0.25 µm ATRA concentration required for differentiation [7]. Additionally, it must be pointed out that early pharmacokinetic data and subsequent large studies that established the role of combination of ATO and ATRA from China have demonstrated the adequacy of an ATRA dose of 25 mg/m^2^ (in contrast to the dose of 45 mg/m^2^ used in the RCT’s) administered continuously for 6–8 weeks in induction and 30 days in each consolidation course [8]. For high-risk APL, the optimal combination, dose, and schedule remain to be defined. The most promising results are shown by a combination of ATO and ATRA combined with anthracycline or gemtuzumab ozagomycin (GO), usually limited to induction) [5, 9].

The limitations of the available real-world data of APL treated with this ATO + ATRA combination are that it is limited by small numbers, intensive use of additional anthracyclines, or being restricted to low and intermediate-risk patients [10–12]. At our tertiary center, we have previously reported on our experience with ATO in APL [13, 14] and the cost-effectiveness of this strategy using generic ATO [15]. Following the publication of the RCTs establishing the efficacy of the combination ATO and ATRA, we changed the standard of care regimens in our institution in January 2015 (summarized in Fig. 1), with some modifications from the published schedules based on our earlier experience and based on the following principle and rationale: (i) use of ATO and ATRA based combination in all patients. (ii) ATO and ATRA alone in patients presenting with WBC counts ≤5 × 10^9^/L. (iii) For those with WBC count >5 but <10 × 10^9^/L we added an abbreviated course of anthracycline in induction alone on day 1 and 2, to reduce problems with leukocytosis, differentiation syndrome and bring about a more rapid correction of the coagulopathy. All these complications, in our setting, contribute significantly to morbidity, early mortality and increased requirement of supportive care and cost of therapy. (iv) For high risk patients with WBC ≥ 10 × 10^9^/L anthracyclines were added in induction and consolidation (day 1 and 2). (v) In induction, for those with a WBC count >5 × 10^9^/L at presentation ATRA was added at full dose on day +7 onwards or once WBC count was <5 × 10^9^/L, whichever was later, in an effort to reduce the risk of hyper-leucocytosis and differentiation syndrome. (vi) Mitoxantrone was the anthracycline used in our protocols mainly because it was the least expensive anthracycline and nothing to suggest that it was inferior to other conventionally used anthracycline. (vii) Post the first consolidation, a bone marrow reverse transcriptase-polymerase chain reaction (RT-PCR) based qualitative report, with a sensitivity of 1 × 10^−4^, was used to decide on need of an additional consolidation with anthracycline. (viii) ATO and ATRA were always administered concurrently, based on proven synergy of this approach and to simplify the schedule for caregivers and patients.

**Fig. 1:**
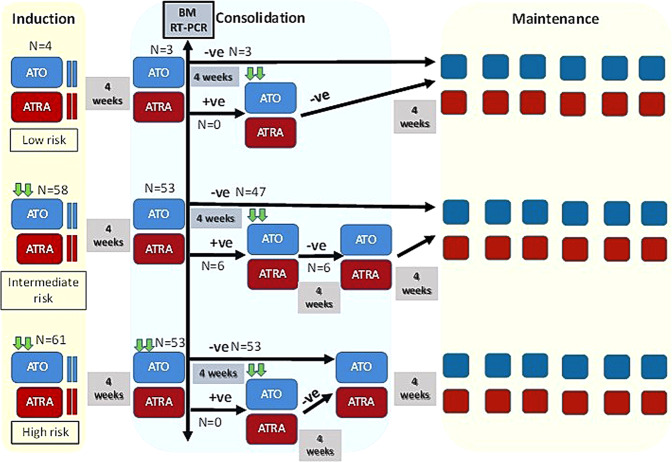
Treatment schedule for newly diagnosed patients with acute promyelocytic leukemia. ATO was sourced from Intas Pharmaceuticals Pvt. Ltd, India, ATRA was sourced from Catalent Germany Eberbach GmbH, Germany, and mitoxantrone was sourced from Neon Laboratories, India. Arsenic trioxide (ATO) was administered at a fixed dose of 10 mg daily intravenously over 3 h (0.15 mg/kg for bodyweight <45 kg). Oral all-trans retinoic acid (ATRA) was administered 45 mg/m^2^ daily in two doses. Mitoxantrone was given at a dose of 10 mg/m^2^ intravenously daily for 2 days. Induction therapy was given for at least 42 days and until achieving complete morphologic remission or a maximum of 60 days. ATRA was started on day 7 of induction for intermediate and high-risk patients or once WBC counts were less than 5 × 10^9^/L, whichever was later. Consolidation blocks were for 28 days each. Bone marrow RT-PCR at the end of the first consolidation was done to document molecular remission. ATRA was added to the low-risk protocol after May 2019 and hence only 4 patients who received both ATRA and ATO concurrently in induction were included in the analysis. In low-risk patients, mitoxantrone was given if WBC count >20 × 10^9^/L in 1st week of treatment, WBC count >50 × 10^9^/L in 2nd week of treatment, differentiation syndrome not resolving with steroids or associated with leukocytosis, or at physician discretion when there was rapid WBC count doubling. Post consolidation concurrent ATO and ATRA were administered for 10 days/month × 6 months (Blue and red squares in maintenance indicate ATO and ATRA respectively), given for 10 days a month for 6 months. Prophylactic CNS directed therapy and steroids were not administered. Patients were classified as low-risk if the peripheral blood white blood cell (WBC) count <5 × 10^9^/L and the platelet count >20 × 10^9^/L and as high-risk if the WBC count was ≥10 × 10^9^/L, all others were considered as intermediate risk. Green arrows: mitoxantrone ATO: arsenic trioxide ATRA: all-trans retinoic acid BM RT-PCR: Bone marrow reverse transcriptase-polymerase chain reaction for *PML-RARA*. −ve: negative +ve: positive.

After the institutional ethics committee’s approval, we performed a retrospective analysis including all consecutive patients diagnosed with APL who presented to our hospital from January 2015 to May 2020. 167 patients with APL were seen at our center during the study period. Of these, we excluded 5 patients who had presented with relapsed disease and 28 patients who were treated with single-agent ATO (Supplementary Fig. 1). The remaining 134 patients were analyzed for real-world clinical outcomes. The data were frozen and analyzed on 1st June 2021, a year after the last patient was included in this study. At a median follow-up of 854 days, there were 2 (1.6%) hematologic relapses and no deaths beyond induction. The 2-year OS and EFS for the intention-to-treat analysis cohort are 91% ± 2.5% and 89.3% ± 2.7%, respectively (Supplementary Fig. 2).

For analysis of regimen safety and efficacy (per protocol analysis), we excluded patients who wished to pursue treatment elsewhere within the first 1 week of treatment (*n* = 7), and patients who had severe infections or life-threatening bleeding at presentation or before therapy initiation and subsequently died (*n* = 4) (Supplementary Table 1: details of excluded patients). Thus, a total of 123 patients with newly diagnosed APL were included for all further analysis. The baseline characteristics, duration of symptoms prior to diagnosis, blood product support, and clinical outcomes of the per-protocol treated cohort (*n* = 123) is summarized in Supplementary Table 2. Eight (6.5%) patients died during induction, and this included 2 patients with high-risk disease who were assigned as dead for the per-protocol analysis since they were discharged against medical advice in a critical clinical condition at days 39 and 59, with fungal brain abscess and cardiac tamponade, respectively and hence were unlikely to have survived. The cause of death for the remaining 6 patients was intracranial bleed (*n* = 2; on day 7 and day 16, respectively) and septic shock (*n* = 4, on days 9, 11, 22, and 42, respectively) (3 each in intermediate-risk and high-risk group). Four (3.3%) patients were discharged against medical advice beyond 2 weeks of treatment but before completing induction and were clinically well at discharge, opting to continue treatment at another center. The remaining 111 (90.2%) patients attained morphologic CR following induction at a median time of 43 days (IQR: 42 to 46.25 days). At presentation, 81 (66%) patients had bleeding symptoms. Major bleeding was noted in 19 patients at presentation (11 had intracranial bleeding, 2 had intra-abdominal bleeding, while 6 had intra-ocular bleeding). One patient had thrombosis (cortical venous sinus thrombosis) at presentation. During induction, nine more patients developed major bleeding (4 had intracranial bleeding, 4 had intra-ocular bleeding, and 1 had pulmonary hemorrhage) at a median of 8 days (IQR: 3 to 9 days). Additionally, four patients developed thrombosis during induction (hepatic venous thrombosis on day 6, cortical venous sinus thrombosis on day 12, and limb deep venous thrombosis on day 14 and 18, respectively). Nineteen (15.4%) patients were clinically diagnosed to have differentiation syndrome during induction. The median day of onset was day 12 (IQR: day 9 to day 15), all patients were treated with steroids, median duration of 3 days (IQR: 2 to 4 days). All except 3 patients developed a neutropenic fever during induction and required antimicrobial therapy. Sixty-seven (55%) patients had at least one documented infection during induction therapy (Supplementary Table 3). During induction, grade 3 hepatotoxicity was noted in 8 (6.5%) patients, 10 (8.1%) patients had ATRA related headache or benign intracranial hypertension of which 5 required transient cessation of ATRA for a median of 5 days (IQR: 4 to 8 days), 11 (8.9%) patients had transient QTc prolongation of which 6 required transient cessation of ATO for a median of 1.5 days (IQR: 1 to 3 days), 17 (13.8%) patients developed symptomatic sensory neuropathy requiring treatment. None required permanent discontinuation of therapy.

Of the 111 patients who attained morphologic CR after induction, 2 patients were lost to follow-up. The remaining 109 patients completed consolidation. Of these, 103 patients were in molecular remission following the first cycle of consolidation therapy, while six patients with intermediate-risk APL required 2 cycles of consolidation to achieve molecular remission. Post consolidation patients received 6 cycles of maintenance therapy (Fig. 1).

At a median follow up of 854 days, there were 2 (1.6%) hematologic relapses and no deaths beyond induction therapy. The 2-year OS and EFS for the per-protocol analysis are 93.4% ± 2.3% and 91.6% ± 2.6%, respectively (Fig. 2A, B). Supplementary Table 4 and Supplementary Fig. 3 show the clinical characteristics and survival of 28 patients with low-risk APL who were excluded from the analysis since they received single agent ATO during induction therapy. Supplementary Table 5 shows the univariate Cox proportional hazard model for OS and EFS for the per-protocol analysis cohort, an activated partial thromboplastin time (APTT) was the only variable which correlated with and adverse impact on OS and EFS in our cohort.

**Fig. 2:**
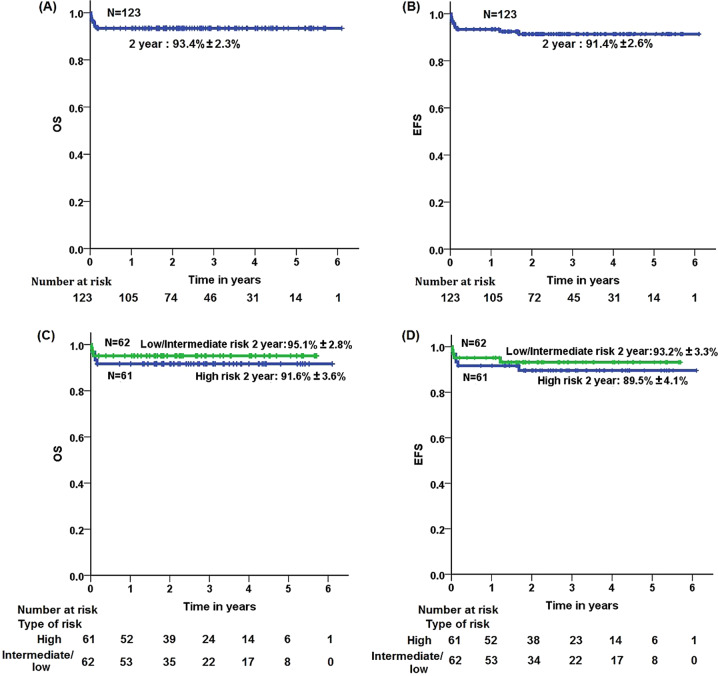
Kaplan Meier estimates of overall and event-free survival. **A** Overall survival for the per-protocol cohort (*N* = 123). **B** Event-free survival for the per-protocol cohort (*N* = 123). **C** Overall survival for patients with low-intermediate risk (*N* = 62) and high risk (*N* = 61) patients with APL. **D** Event-free survival for patients with low-intermediate risk (*N* = 62) and high risk (*N* = 61) patients.

This study illustrates the efficacy of the combination of ATRA and ATO even outside a clinical trial setting and especially its efficacy in combination with anthracycline in the high-risk subset. While a formal cost analysis, similar to what was reported earlier with single-agent generic ATO [15], was not done, our preliminary analysis does not suggest that this combination approach adds significantly to the cost. The total direct hospital costs incurred for the treatment within 1 year of diagnosis was approximately USD 6400 per patient. While it will not be possible to conclude from this study that concurrent administration of ATO and ATRA contributed to improved efficacy and clinical outcomes, it would be reasonable to conclude that this approach is not detrimental to efficacy, as has already been demonstrated by the earlier reported studies from China, and is probably easier to administer and monitor for patients and caregivers. There is scope to further explore the optimal dose and schedule of these agents in the clinical trial setting, specifically the reduction in the dose of ATRA and optimizing the use of anthracycline based on a post-consolidation BM RT-PCR, as was done in this study.

Our data illustrate the possibility of delivering the highest standard of care at a universally affordable cost to treat a leukemia and meets the aspiration, for the first time, of global equitable health care delivery in leukemia.

## Supplementary information

Supplementary

## References

[1] Lo-Coco F, Avvisati G, Vignetti M, Thiede C, Orlando SM, Iacobelli S (2013). Retinoic acid and arsenic trioxide for acute promyelocytic leukemia. N. Engl J Med.

[2] Burnett AK, Russell NH, Hills RK, Bowen D, Kell J, Knapper S (2015). Arsenic trioxide and all-trans retinoic acid treatment for acute promyelocytic leukaemia in all risk groups (AML17): results of a randomised, controlled, phase 3 trial. Lancet Oncol.

[3] Sanz MA, Fenaux P, Tallman MS, Estey EH, Lowenberg B, Naoe T (2019). Management of acute promyelocytic leukemia: updated recommendations from an expert panel of the European LeukemiaNet. Blood.

[4] Lallemand-Breitenbach V, Guillemin MC, Janin A, Daniel MT, Degos L, Kogan SC (1999). Retinoic acid and arsenic synergize to eradicate leukemic cells in a mouse model of acute promyelocytic leukemia. J Exp Med.

[5] Ravandi F, Estey E, Jones D, Faderl S, O’Brien S, Fiorentino J (2009). Effective treatment of acute promyelocytic leukemia with all-trans-retinoic acid, arsenic trioxide, and gemtuzumab ozogamicin. J Clin Oncol.

[6] Ozpolat B, Lopez-Berestein G, Adamson P, Fu CJ, Williams AH (2003). Pharmacokinetics of intravenously administered liposomal all-trans-retinoic acid (ATRA) and orally administered ATRA in healthy volunteers. J Pharm Pharm Sci.

[7] Tasseff R, Jensen HA, Congleton J, Dai D, Rogers KV, Sagar A (2017). An effective model of the retinoic acid induced HL-60 differentiation program. Sci Rep..

[8] Shen ZX, Shi ZZ, Fang J, Gu BW, Li JM, Zhu YM (2004). All-trans retinoic acid/As2O3 combination yields a high quality remission and survival in newly diagnosed acute promyelocytic leukemia. Proc Natl Acad Sci USA.

[9] Iland HJ, Collins M, Bradstock K, Supple SG, Catalano A, Hertzberg M (2015). Use of arsenic trioxide in remission induction and consolidation therapy for acute promyelocytic leukaemia in the Australasian Leukaemia and Lymphoma Group (ALLG) APML4 study: a non-randomised phase 2 trial. Lancet Haematol.

[10] Kayser S, Schlenk RF, Lebon D, Carre M, Gotze KS, Stolzel F, et al. Characteristics and outcome of patients with low-/intermediate-risk acute promyelocytic leukemia treated with arsenic trioxide—an international collaborative study. Haematologica. 2021;106:3100–106. 10.3324/haematol.2021.278722 PMC8634174 34047178

[11] Yang S, Ma R, Yuan X, Jiang L, Shi J, Yang J (2020). Improved outcomes of all-trans-retinoic acid and arsenic trioxide plus idarubicin as a frontline treatment in adult patients with acute promyelocytic leukemia. Clin Lymphoma Myeloma Leuk.

[12] Danthala M, Golamari KR, Seshachalam A, Mikkilineni A, Chappidi S, Mekala MB (2020). Walking a tightrope: dosage modifications and treatment outcomes of all-trans-retinoic acid, arsenic trioxide, and daunorubicin for high-risk acute promyelocytic leukemia. JCO Glob Oncol..

[13] Mathews V, George B, Lakshmi KM, Viswabandya A, Bajel A, Balasubramanian P (2006). Single-agent arsenic trioxide in the treatment of newly diagnosed acute promyelocytic leukemia: durable remissions with minimal toxicity. Blood..

[14] Mathews V, George B, Chendamarai E, Lakshmi KM, Desire S, Balasubramanian P (2010). Single-agent arsenic trioxide in the treatment of newly diagnosed acute promyelocytic leukemia: long-term follow-up data. J Clin Oncol.

[15] Bankar A, Korula A, Kulkarni UP, Devasia AJ, Na F, Lionel S (2020). Resource utilization and cost effectiveness of treating acute promyelocytic leukaemia using generic arsenic trioxide. Br J Haematol.

