# Supplementary material for: Real world data with concurrent retinoic acid and arsenic trioxide for the treatment of acute promyelocytic leukemia
Source: Blood Cancer J. 2022 Jan 31;12(1):22. doi: 10.1038/s41408-022-00619-3 (PMC8803919; doi:10.1038/s41408-022-00619-3)
Supplement: Supplementary file 1 — Supplementary [file 41408_2022_619_MOESM1_ESM.docx]

**Supplementary**

# Real World Data with Concurrent Retinoic acid and Arsenic trioxide for the Treatment of Acute Promyelocytic Leukemia

Uday P. Kulkarni^1^, Sushil Selvarajan^1^, Sharon Lionel^1^, Mithun A. Prakash^1^, Hamenth Kumar Palani^1^, Nithya Balasundaram^1^, Arvind Venkat^1^, Anu Korula^1^, Anup J. Devasia^1^, Fouzia NA^1^, Nancy Beryl Janet^1^, Sukesh Chandran Nair^2^ Aby Abraham^1^,  Thenmozhi Mani^3^, Jeyaseelan Lakshmanan^3^, Poonkuzhali Balasubramanian^1^, Biju George^1^, Vikram Mathews^1^

^1^ Department of Haematology, Christian Medical College, Vellore, India

^2^Department of Immunohaematology and Transfusion Medicine, Christian Medical College, Vellore, India.

^3^ Department of Biostatistics, Christian Medical College, Vellore, India.

**Supplementary methods:**

1. Low-risk protocol
2. Intermediate risk protocol
3. High-risk protocol
4. Supportive care protocol

**Supplementary results:**

**Supplementary methods:**

Vellore Low-Risk Group Protocol

**All age groups**

**Confirm diagnosis by FISH / RT-PCR**

**Pregnancy is a relative contra-indication
Low risk group: defined as WBC < 5000/mm^3^ AND Platelet > 20,000/mm^3^**

**INDUCTION:**

Injection ATO: 10 mg / day IV over 3 hours (<45 kg – 0.15 mg/kg)

Cap ATRA: 45mg/m^2^/day PO in two divided doses

(Start both on day 1)

Minimum duration of 6 weeks.

Maximum duration of 60 days

Indications for anthracycline in induction (Mitoxantrone 10mg/m2/day x 2 days)

1. Within First week leukocytosis : >20,000/mm3 after starting treatment
2. Within Second week leukocytosis: >50,000/mm3 after starting treatment
3. Differentiation syndrome: : not resolving with steroids or associated

with onset of leukocytosis

1. Not fulfilling the above criteria but WBC count rapid doubling

(Treating physician discretion)

Document complete remission (not before completion of week 6 or day 42 of ATO)

Bone marrow to be done when peripheral blood counts are normal (ANC >1000/mm^3^, Platelet >100,000/mm^3^). If bone marrow is not in remission, continue ATO and repeat bone marrow at weekly intervals till hematological remission is documented.

**4 week break**

**CONSOLIDATION:**

Injection ATO : 10 mg / day / IV Day 1 - 28

(<45 kg – 0.15 mg/kg) (total 28 doses, break permitted for Sundays)

Cap ATRA 45mg/m^2^/day PO in two divided doses Day 1- 28

**4 weeks break**

**Maintenance:**

Injection ATO 10 mg / day / IV (<45 kg – 0.15 mg/kg) 10 days/month x 6 months

Cap ATRA 45mg/m^2^/day PO in two divided doses 10 days/month x 6 months

**MRD monitoring:** Done once after consolidation completed **BM-RT: PCR**

**If this is negative,** no further MRD monitoring is required.

**If positive,** administer **Consolidation I** as per High-risk protocol. If BM-RTPCR is negative after this (after 4-week course), follow maintenance as per high-risk protocol (same monitoring strategy as for high risk). If still positive individualize – discuss with treating physician.

Review once in 3 months for 2 years and then once in 6 months for 3 years

At review: Peripheral blood for CBC, LFT, Creatinine, RBS

Other tests as indicated

Vellore Intermediate Risk Group Protocol

**All age groups**

**Confirm diagnosis by FISH / RT-PCR**

**Pregnancy is a relative contra-indication
Intermediate risk group: defined as**

**WBC ≥ 5000/mm^3^ but <10,000/mm^3^**

**OR**

**WBC < 5000/mm^3^ but Platelet count ≤20,000/mm^3^**

**INDUCTION:**

1. Injection Mitoxantrone: 10 mg/m^2^/IV once a day Day 1 and 2
2. Injection ATO : 10 mg / day IV over 3 hours (<45 kg – 0.15 mg/kg)] *
3. Cap ATRA 45mg/m^2^/day PO in two divided doses^ ]*

* Minimum duration 42 days. Maximum duration in induction 60 days

^ Start earliest day 7 and after WBC count <5000/mm^3^ (both criteria to be fulfilled)

Document complete remission (not before completion of week 6 or day 42 of ATO)

Bone marrow to be done: only when the peripheral blood counts are normal (ANC >1000/mm^3^, Platelet >100,000/mm^3^). If bone marrow is not in remission, continue ATO and ATRA and repeat bone marrow at weekly intervals till hematological remission is documented.

**4 week break**

**CONSOLIDATION:**

1. Injection ATO : 10 mg / day / IV (<45 kg – 0.15 mg/kg) Day 1 – 28
2. Cap ATRA 45mg/m^2^/day PO in two divided doses Day 1 - 28

**4 weeks break**

**Maintenance:**

1. Injection ATO : 10 mg/day/IV (<45 kg – 0.15 mg/kg) 10 days/month x 6 months
2. Cap ATRA 45mg/m^2^/day PO in two divided doses 10 days/month x 6 months

**MRD monitoring:** Done once after consolidation completed **BM-RT: PCR**

**If negative:** continue the same protocol

**If positive:** to move to high-risk protocol starting from Consolidation 1

Subsequently, **PB RT: PCR** prior to maintenance 1,3 and 6

Post maintenance **PB RT: PCR** once in 3 months x 2 years (extend another year if any reading was positive in this period but not fulfilling criteria for molecular relapse, i.e., at least 2 consecutive RT:PCR positive at 3-4 week intervals

Beyond two years review once in 6 months x 3 years

At review: PB for CBC, LFT, Creatinine, RBS

Other tests as indicated

Vellore High Risk Group Protocol

**All age groups**

**Confirm diagnosis by FISH / RT-PCR**

**Pregnancy is a relative contra-indication
High-risk group: defined as WBC ≥ 10,000/mm^3^**

**INDUCTION:**

1. Injection Mitoxantrone: 10 mg/m^2^/IV once a day Day 1 and 2
2. Injection ATO : 10 mg / day IV over 3 hours (<45 kg – 0.15 mg/kg)] *
3. Cap ATRA 45mg/m^2^/day PO in two divided doses^ ]*

* Minimum duration 42 days. Maximum duration in induction 60 days

^ Start earliest day 7 or after WBC count <5000/mm^3^ (both criteria to be fulfilled)

Document complete remission (not before completion of week 6 or day 42 of ATO)

Bone marrow: to be done when peripheral blood counts are normal (ANC >1000/mm^3^, Platelet >100,000/mm^3^). If bone marrow is not in remission, continue ATO and ATRA and repeat bone marrow at weekly intervals till remission is documented.

**4 week break**

**CONSOLIDATION I:**

1. Injection Mitoxantrone : 10 mg/m^2^/IV once a day Day 1 and 2
2. Injection ATO : 10 mg / day / IV (<45 kg – 0.15 mg/kg) Day 1 – 28
3. Cap ATRA 45mg/m^2^/day PO in two divided doses Day 1 - 28

**4 weeks break**

**CONSOLIDATION II:**

1. Injection ATO : 10 mg / day / IV (<45 kg – 0.15 mg/kg) Day 1 – 28
2. Cap ATRA 45mg/m^2^/day PO in two divided doses Day 1 - 28

**4 weeks break**

**Maintenance:**

1. Injection ATO : 10 mg/day/IV (<45 kg – 0.15 mg/kg) 10 days/month x 6 months
2. Cap ATRA 45mg/m^2^/day PO in two divided doses 10 days/month x 6 months^

^ To be administered concurrently

**MRD monitoring:** Done once after consolidation completed **BM-RT: PCR**

Subsequently, PB RT: PCR prior to maintenance 1,3 and 6

Post maintenance PB RT: PCR once in 3 months x 3 years

Subsequent follow up once in 6 months x 2 years

At review: PB for CBC, LFT, Creatinine, RBS

Other tests as indicated

**Protocol for supportive therapy and management of the differentiation syndrome**

**During induction:** Monitor counts daily till stable, followed by 2 to 3 times/week till recovery from cytopenia.

Monitor PT, APTT, and fibrinogen at admission and daily for the first week. The second week, if stable, parameters normal and no bleeding: monitor on alternate days (daily if abnormal, symptoms of bleeding).

If PT / APTT abnormal ± active bleeding transfuse fresh frozen plasma [FFP] 15 ml/kg, if active bleeding repeat till normal. If Fibrinogen <140mg% transfuse 1-2 units of cryoprecipitate / 10kg and recheck fibrinogen the next day (more frequently if active bleed).

Transfuse Platelet concentrates to maintain platelet count >30,000/mm^3^ in the absence of bleeding and >50,000/mm^3^ in the presence of bleeding.

Day 1 if platelet count <30,000/mm3 transfuse 2 PRC twice daily, after first infusion check platelet count after 1 - 4 hours, based on this value increase PRC transfusion support to achieve the above target.

High clinical suspicion of intracranial bleeding is to be maintained till recovery from cytopenia. If the patient complains of headache, transfuse 4 PRC urgently and an urgent CT scan must be done [C-] (DO NOT WAIT FOR CT SCAN TO BE DONE OR REPORTED. If a bleed is present, transfusion support should be increased to target a platelet count of 50,000/mm3 and transfuse FFP’s/Cryoprecipitate to maintain normal hemostatic parameters.

Leukocytosis can occur with this therapy which can result in DIC, leukostasis, and a differentiation syndrome. Hydroxyurea will be administered to control leukocytosis if required. A total white cell count will be done on alternate days unless otherwise indicated and hydroxyurea administered according to the following guidelines.

**WBC > 5000 - 10000/mm^3^ Hydroxyurea 500 mg OD**

**WBC > 10000 – 15000/mm^3^ Hydroxyurea 500 mg BD**

**WBC > 15000 – 20000/mm3 Hydroxyurea 500 mg TID**

**WBC > 20000 – 50000/mm3 Hydroxyurea 500 mg QID**

**WBC > 50000/mm3 Hydroxyurea 1.0 gm QID**

Electrolytes: Monitor alternate days (more frequently if required). Maintain K+>4mEq/Lt and Mg+ >2mg%

ECG: Monitor once a week during induction only (more frequently if required). Consider stopping ATO if corrected QTc>500msec (Note: Bazetts formula overcorrects in the setting of tachycardia due to any etiology. More significant if Qtc prolonged with HR < 100 / min)

**Differentiation Syndrome:**

Clinical diagnosis - there is no definite diagnostic criteria.

Major manifestations [>10% incidence] include:

-Respiratory distress -Hypotension

-Fever

-Pulmonary oedema, pulmonary infiltrates -Headache

-Pleural or pericardial effusion -Congestive cardiac failure

-Acute renal failure

#### **Treatment of Differentiation syndrome**

### Stop ATO, ATRA if severe

Inj Dexamethasone 10 mg IV twice daily 3 - 5 days.

If the syndrome resolves, re-institute ATO at 50% of the initial dose and escalate to the full dose in 3 - 5 days if the syndrome does not recur.

**Supplementary Results:**

**Supplementary Figure 1: Flowchart delineating the patients included in the study.**


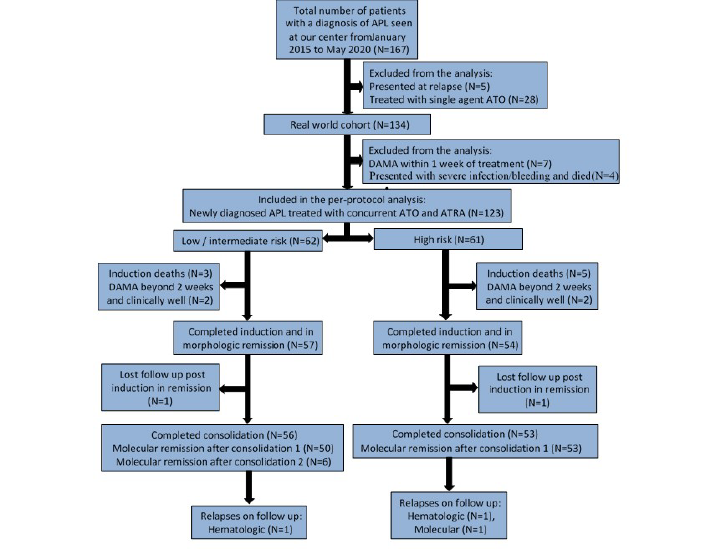


APL: acute promyelocytic leukemia

DAMA: discharge against medical advice

ATO: arsenic trioxide

ATRA: all-trans retinoic acid

**Supplementary figure 2: Kaplan Meier estimates of overall and event-free survival** for the real world cohort of all newly diagnosed patients with APL offered combined ATO and ATRA therapy at our center during the study period (N=134)


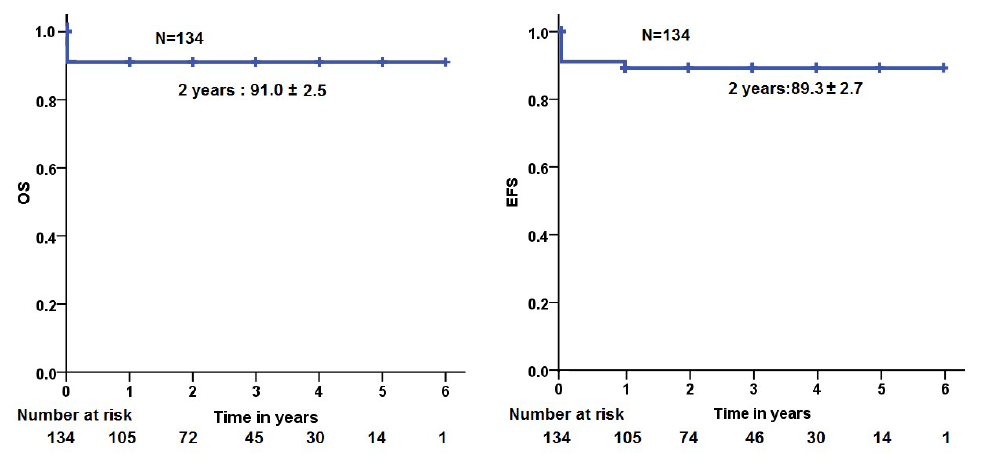

**Supplementary Figure 3:** Kaplan Meier estimates of (A) Overall survival and (B) Event-free survival of the 28 patients with low-risk acute promyelocytic leukemia who received single-agent arsenic trioxide during induction

**Supplementary Table 1:** Clinical details of patients who were excluded from the analysis of treatment efficacy (N = 11)

| **UPN** | **Age in years** | **Sex** | **Symptom duration** | **Clinical details at presentation** | **APL risk category** | **Total days of admission** | **Status at last follow-up** |
| --- | --- | --- | --- | --- | --- | --- | --- |
| 8 | 10 | M | 2 weeks | Fever since 2 weeks and gum bleeding for 1 week. Systemic examination unremarkable except for pallor | LR | 3 | DAMA in a stable clinical condition |
| 36 | 23 | F | 4 weeks | Underwent MTP 2 days prior elsewhere, presented with fatigue, vomiting and left loin pain; had sepsis with ARDS (73% saturation at presentation) | HR | 2 | DAMA in critical condition |
| 59 | 42 | M | 2 weeks | Presented with fever, gum bleeding, bleeding per rectum and breathlessness; had septic shock (BP 86/60 mmHg, 88% saturation) | HR | 5 | Died of IC bleed |
| 66 | 25 | M | 2 weeks | Received 2 packed cells and 10 platelet rich concentrates elsewhere, presented with fever and gum bleeding, developed IC bleed within 24 hours of admission and prior to confirmation of diagnosis | HR | 6 | DAMA in critical condition |
| 70 | 52 | M | 1 week | Received 6 platelet rich concentrates elsewhere, presented with gum bleeding and petechiae; developed pulmonary hemorrhage within 48 hours of admission and prior to confirmation of diagnosis | HR | 6 | DAMA in critical condition |
| 94 | 36 | M | 2 weeks | Fever associated with headache. Systemic examination was unremarkable. Brain imaging ruled out intracranial bleeding | HR | 2 | DAMA in a stable clinical condition |
| 97 | 17 | M | 1 week | Penile swelling associated with fever, dysuria and skin rash. Systmic examination showed multiple erythematous vesicular skin lesions over the body along with paraphymosis | IR | 3 | DAMA in a stable clinical condition |
| 115 | 20 | M | 1 week | Presented with fever and headache; had low sensorium on admission with MRI brain showing bilateral cerebellar infarcts with microhemorrhages | HR | 12 | Died of IC bleed |
| 129 | 54 | M | 2 weeks | Admitted in ICU elsewhere for 1 week and treated with GCSF, antibiotics and 12 platelet rich concentrates, presented to us with fever, and loose stools, noted to have bilateral pneumonia (86% saturation) | HR | 5 | DAMA in critical condition |
| 139 | 45 | M | 4 weeks | Presented with fever, hematuria, hemoptysis, bleeding gums and reduced vision - finger counting at 2 feet, developed pulmonary hemorrhage within 24 hours of admission | HR | 4 | Died of pulmonary hemorrhage |
| 165 | 42 | M | 2 weeks | Presented with fever and breathlessness, had sepsis with ARDS (80% saturation) | HR | 6 | Died of enterococcal sepsis |

UPN = unique patient identification number, F = female, M = male, MTP = medical termination of pregnancy, ARDS = acute respiratory distress syndrome, BP = blood pressure, MRI = magnetic resonance imaging, ICU = intensive care unit, GCSF = granulocyte colony stimulating factor, APL = acute promyelocytic leukemia, HR = high risk, IR = intermediate risk, LR = low risk, DAMA = discharge against medical advice, IC = intracranial)

**Supplementary Table 2:** Characteristics of the per-protocol cohort of patients with newly diagnosed acute promyelocytic leukemia (N=123)

| **Variables** | **Mean ± SD / N (%) / Median (range)** |
| --- | --- |
| **Clinical parameters** | **-** |
| Age (in years) | 33.7 **±** 13.6 |
| Female sex | 46 (37) |
| Duration of symptoms (in days) | 14 (7-28) |
| Risk group | **-** |
| Low | 4 (3.3) |
| Intermediate | 58 (47.2) |
| High | 61 (49.6) |
| **Laboratory parameters at presentation** | - |
| Hemoglobin (in g/dL) | 7.8 **±** 2.3 |
| Total white blood cell counts (x 10^9/L) | 9.2 (2.8-34.7) |
| Platelet counts (x 10^9/L) | 16 (10-26) |
| Blasts and promyelocytes in peripheral blood (%) | 71 (35-89) |
| Serum creatinine (in mg/dL) | 0.8 (0.6-1.0) |
| Prothrombin time (in s) | 14.4 **±** 3.1 |
| Activated partial thromboplastin time (in s) | 34.7 **±** 8.0 |
| Fibrinogen (in mg/dL) | 172 (109-273) |
| Serum lactate dehydrogenase (U/L) | 757 (495-1166) |
| Total bilirubin (in mg/dL) | 0.8 (0.5-0.9) |
| Serum albumin (in g/dL) | 4.3 (3.8-4.7) |
| Serum alanine aminotransferase (in U/L) | 24 (15-43) |
| Serum alkaline phosphatase (in U/L) | 72.5 (58-108.8) |
| **Blood product support during induction** | - |
| Packed red cell concentrates | 5 (3-6.5) |
| Fresh frozen plasma | 10 (3-30) |
| Platelet rich concentrates | 40 (23-55) |
| Cryoprecipitate | 6 (0-18) |
| **Days to hematologic complete remission (CR)** | 43 (42-46.3) |
| **Follow up time (in days)** | 854 (587-1460) |
| **Death** | 8 (6.5) |
| **Hematologic relapse** | 2 (1.6) |
| **Cumulative dose of arsenic trioxide used (in mg)** | 1350 (1289-1590) |

**Supplementary Table 3:** Documented infective episodes during induction therapy

| **Site of infection** | **Gram-positive** | **Gram-negative** | **Mycobacterial** | **Viral** | **Fungal** |
| --- | --- | --- | --- | --- | --- |
| Blood | CONS – 23, MSSA – 1, Enterococci – 2 | NFGNB – 5 (CRO - 1),  Pseudomonas – 1,  E.coli – 2 (ESBL – 1),  Enterobacter – 1 | Mycobacterium abscessus – 1 | Hepatitis B Virus – 1,  Dengue – 1 | Candida tropicalis – 2 Candida parapsilosis – 1, Candida albicans – 1 |
| Central venous catheter tip | MSSA – 1 | Acinetobacter baumannii – 1,  Klebsiella pneumoniae – 1 (CRO) | - | - | - |
| Skin and soft tissue | MRSA – 2, Enterococci – 3 (VRE - 1) | E.coli – 1,  NFGNB – 1 (CRO) | - | Herpes simplex virus– 2, Varicella zoster virus – 1 | Aspergillus fumigatus, Absidia corymbifera – 1 |
| Respiratory tract | MRSA – 1 | Pseudomonas – 1,  H. Influenzae – 1,  E.coli – 1 (CRO)  Klebsiella pneumoniae – 3, Strenotrophomonas maltophila – 1, Acinetobacter baumannii – 3 (of which 2 were CRO) ,  Klebsiella pneumoniae – 1 (CRO) NFGNB – 1 (CRO). | - | Rhinovirus – 1, Enterovirus – 1, Influenza B – 3 | Fungal pneumonia  probable – 1  possible – 1 |
| Gastrointestinal tract | Clostridium difficile – 1 | - | - | - | Candida – 1 |
| Urinary tract | Enterococci - 8 | E.coli – 3 (CRO - 1, ESBL - 1), Klebsiella pneumoniae – 1 | - | - | - |

CONS = coagulase negative Staphylococci, MSSA = methicillin sensitive Staphylococcus Aureus, MRSA = methicillin resistant Staphylococcus Aureus, VRE = vancomycin resistant enterococci, NFGNB = non-fermenting gram negative bacteria, E. coli = Escherichia coli, CRO = carbapenem resistant organism, ESBL = extended spectrum beta lactamase producing, H.influenza = Haemophilus influenza

**Supplementary Table 4:** Characteristics of 28 patients with low-risk acute promyelocytic leukemia who were excluded as they received single-agent arsenic trioxide during induction

| **Variables** | **Mean ± SD / N (%) / Median (range)** |
| --- | --- |
| **Clinical parameters** | - |
| Age (in years) | 35.3 **±** 15.6 |
| Female sex | 13 (46.4) |
| Duration of symptoms (in days) | 14 (14-28) |
| Major bleeding | 0 (0) |
| Major thrombosis | 3 (10.7) |
| Deep veins of the limb | 1 |
| Limb artery | 1 |
| Stroke | 1 |
| Differentiation syndrome | 6 (21.4) |
| **Laboratory parameters at presentation** | - |
| Hemoglobin (in g/dL) | 8.1 **±** 2.8 |
| Total white blood cell counts (x 10^9/L) | 1.3 (0.8-2.8) |
| Platelet counts (x 10^9/L) | 39 (26-55) |
| Blasts and promyelocytes in peripheral blood (%) | 14 (0-43) |
| Serum creatinine (in mg/dL) | 0.8 (0.6-0.9) |
| Prothrombin time (in s) | 13.4 **±** 5.0 |
| Activated partial thromboplastin time (in s) | 32.9 **±** 5.0 |
| Fibrinogen (in mg/dL) | 293 (199-406) |
| Serum lactate dehydrogenase (U/L) | 464 (372-575) |
| Total bilirubin (in mg/dL) | 0.5 (0.4-0.7) |
| Serum albumin (in g/dL) | 4.0 (3.6-4.6) |
| Serum alanine aminotransferase (in U/L) | 21 (10-45) |
| Serum alkaline phosphatase (in U/L) | 75 (59-90) |
| **Blood product support during induction** | - |
| Packed red cell concentrates | 3 (2-5) |
| Fresh frozen plasma | 2 (0-17) |
| Platelet rich concentrates | 12 (0-27.5) |
| **Days to complete hematologic remission (CR)** | 46.5 (43-52) |
| Absolute neutrophil count at CR ( in 10^9/L) | 1.2 (1.0-1.7) |
| Patients requiring hydroxyurea during induction | 12 (42.9) |
| **Death** | 3 (10.7) |
| Hematologic relapse | 5 (17.9) |
| The cumulative dose of arsenic trioxide used (in mg) | 1370 (1320-1473) |

**Supplementary Table 5:** Univariate Cox Proportional hazard model for predictors of overall and event-free survival in the per-protocol cohort (N=123)

| **Variable** | **Overall survival** | | | **Event-free survival** | | |
| --- | --- | --- | --- | --- | --- | --- |
|  | **HR** | **95% - CI** | **P value** | **HR** | **95% - CI** | **P value** |
| **Age** (in years) | 1.01 | 0.96 – 1.07 | 0.672 | 1.02 | 0.97 – 1.07 | 0.497 |
| **Sex:Male** | 1.72 | 0.35 – 8.54 | 0.505 | 2.37 | 0.50 – 11.15 | 0.276 |
| **Duration of symptoms** (in days) | 0.95 | 0.88 – 1.04 | 0.274 | 0.97 | 0.90 – 1.03 | 0.315 |
| **Hemoglobin** (g/dL) | 1.03 | 0.76 – 1.40 | 0.846 | 1.06 | 0.80 – 1.39 | 0.691 |
| **Total white blood cell count** ( x 10^9/L) | 1.00 | 0.98 – 1.02 | 0.838 | 0.99 | 0.97 – 1.02 | 0.605 |
| **Platelet count** ( x 10^9/L) | 1.01 | 0.99 – 1.02 | 0.609 | 1.00 | 0.98 – 1.02 | 0.866 |
| **Peripheral blood blasts and promyelocytes** (%) | 1.00 | 0.98 – 1.03 | 0.832 | 1.00 | 0.98 – 1.02 | 0.873 |
| **Prothrombin time** (in s) | 0.97 | 0.76 – 1.22 | 0.767 | 0.99 | 0.81 – 1.22 | 0.968 |
| **Activated partial thromboplastin time** (in s) | 1.08 | 1.04 – 1.13 | **<0.001** | 1.08 | 1.03 – 1.13 | **0.001** |
| **Serum creatinine** (in mg%) | 0.91 | 0.16 – 7.19 | 0.927 | 1.52 | 0.40 – 5.81 | 0.543 |
| **Fibrinogen** (in mg%) | 0.99 | 0.99 – 1.00 | 0.621 | 1.00 | 0.99 – 1.00 | 0.372 |
| **Bone marrow blasts and promyelocytes** (%) | 1.04 | 0.96 – 1.12 | 0.358 | 1.03 | 0.97 – 1.10 | 0.340 |
| **Serum lactate dehydrogenase** (in U/L) | 1.00 | 0.99– 1.00 | 0.612 | 1.00 | 0.99 – 1.00 | 0.651 |
| **Total bilirubin** (in mg/dL) | 1.32 | 0.40 – 4.36 | 0.645 | 1.60 | 0.59 – 4.30 | 0.355 |
| **Serum albumin** (in g/dL) | 1.05 | 0.33 – 3.34 | 0.931 | 1.03 | 0.37 – 2.89 | 0.949 |
| **Serum alanine aminotransferase** (in U/L) | 1.00 | 0.99 – 1.01 | 0.479 | 1.00 | 0.99 – 1.01 | 0.483 |
| **Serum alkaline phosphatase** (in U/L) | 1.00 | 0.99 – 1.01 | 0.932 | 0.99 | 0.98 – 1.01 | 0.656 |
| **Type of Risk** | - | - | - | - | - | - |
| High | 1.69 | 0.41 – 7.10 | 0.47 | 1.51 | 0.42 – 5.34 | 0.525 |
| Low-intermediate | 1 | - | - | 1 | - | - |
